# Complement networks in gene-edited pig xenotransplantation: enhancing transplant success and addressing organ shortage

**DOI:** 10.1186/s12967-024-05136-4

**Published:** 2024-04-02

**Authors:** Yinglin Yuan, Yuanyuan Cui, Dayue Zhao, Yuan Yuan, Yanshuang Zhao, Danni Li, Xiaomei Jiang, Gaoping Zhao

**Affiliations:** 1 Department of Gastrointestinal Surgery, Sichuan Academy of Medical Sciences & Sichuan Provincial People’s Hospital, School of Medicine, University of Electronic Science and Technology of China, Chengdu, China; 2 Clinical Immunology Translational Medicine Key Laboratory of Sichuan Province, Sichuan Provincial People’s Hospital, University of Electronic Science and Technology of China, Chengdu, China; 3 Department of Pharmacy, The People’s Hospital of Leshan, Leshan, China; 4 Department of Pharmacy, Longquanyi District of Chengdu Maternity & Child Health Care Hospital, Chengdu, China

**Keywords:** Xenotransplantation, Complement systems, Genetically modified pigs, Clinical trials, α-1,3-galactosyltransferase gene-knockout

## Abstract

The shortage of organs for transplantation emphasizes the urgent need for alternative solutions. Xenotransplantation has emerged as a promising option due to the greater availability of donor organs. However, significant hurdles such as hyperacute rejection and organ ischemia–reperfusion injury pose major challenges, largely orchestrated by the complement system, and activated immune responses. The complement system, a pivotal component of innate immunity, acts as a natural barrier for xenotransplantation. To address the challenges of immune rejection, gene-edited pigs have become a focal point, aiming to shield donor organs from human immune responses and enhance the overall success of xenotransplantation. This comprehensive review aims to illuminate strategies for regulating complement networks to optimize the efficacy of gene-edited pig xenotransplantation. We begin by exploring the impact of the complement system on the effectiveness of xenotransplantation. Subsequently, we delve into the evaluation of key complement regulators specific to gene-edited pigs. To further understand the status of xenotransplantation, we discuss preclinical studies that utilize gene-edited pigs as a viable source of organs. These investigations provide valuable insights into the feasibility and potential success of xenotransplantation, offering a bridge between scientific advancements and clinical application.

## Introduction

Transplantation is a crucial strategy for addressing end-stage organ failure. However, the persistently low supply of donated human organs has resulted in a growing demand that far exceeds the available supply. In the U.S. alone, with 103,327 individuals awaiting organ transplants, only 42,000 transplants were conducted in 2022, leading to a tragic daily toll of 17 lives lost while awaiting organs [1]. Recognizing the striking similarities in size and various biological aspects between porcine and human organs, pigs have emerged as prime candidates for xenotransplantation. Their potential is further amplified through genetic engineering, enabling pigs to serve as optimized sources for cells, tissues, and organs in transplantation [2]. Recent strides in genome editing have significantly propelled advancements in this field, but graft rejection remains a pressing problem.

A critical factor contributing to xenograft failure is the activation of the complement system, resulting in hyperacute rejection, ischemia–reperfusion injury, coagulation disorders, and related inflammatory responses. Consequently, comprehending and mitigating immune rejection triggered by the complement system is paramount. In this article, we summarize the evolving landscape of complement in the context of xenotransplantation, explore preclinical applications involving gene-edited pigs related to complement, and outline strategies for regulating complement networks to enhance the efficacy of xenotransplantation. By navigating the intricate interplay between genetic engineering and complement biology, this review aims to contribute to the ongoing dialogue regarding xenotransplantation's potential to address the growing disparity between organ supply and demand.

## Three paths of complement activation

**Fig. 1:**
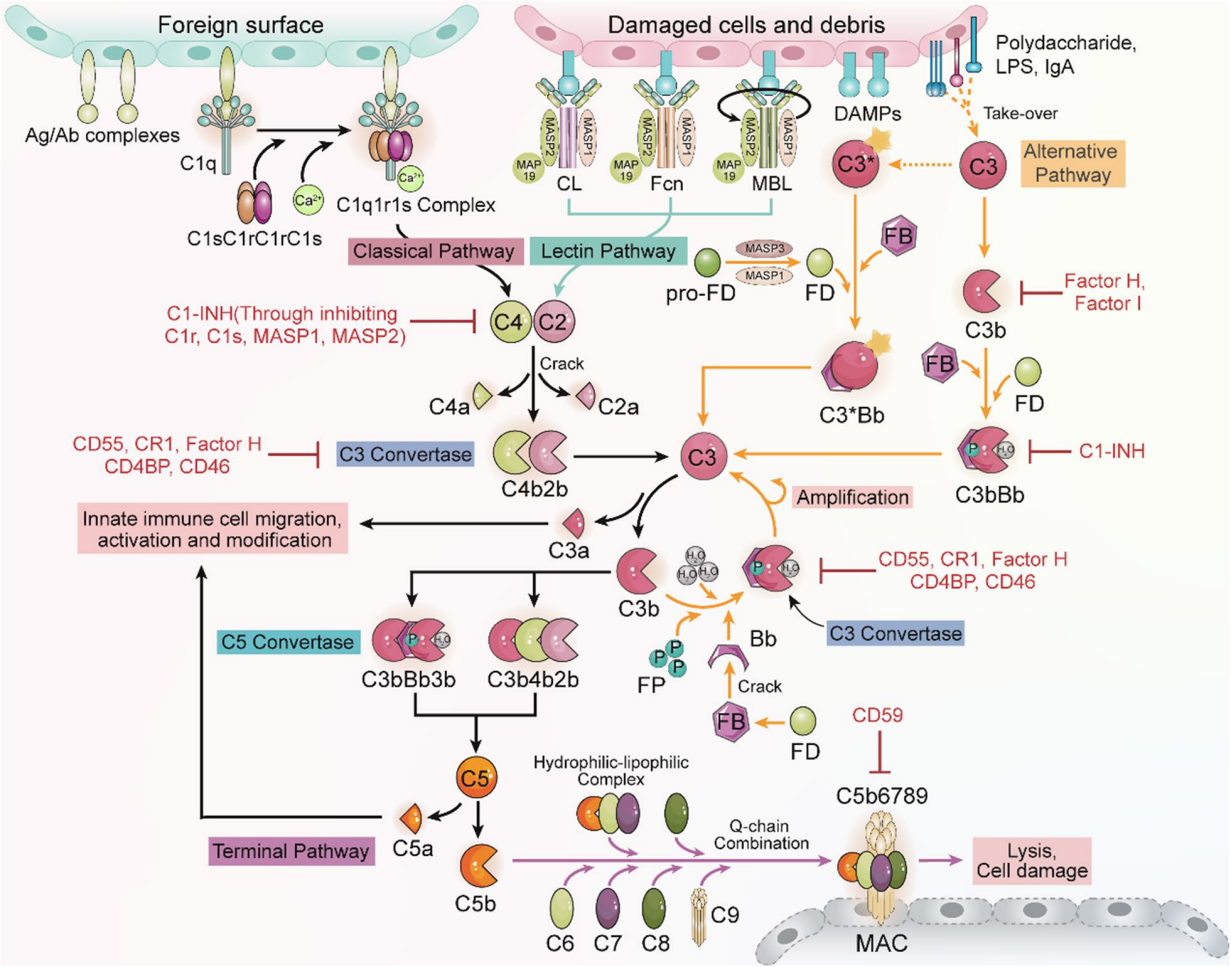
Simplified overview of the active complement cascade. Foreign surface-bound antigen–antibody (Ag/Ab) complexes initiate the classic pathway, while polysaccharides, lipopolysaccharides, and/or IgA activate the alternative complement pathway. Damage-associated molecular patterns (*DAMPs*) like mannose-binding lectin (*MBL*), ficolins (*Fcns*), and certain collections (*CLs*) can directly trigger the classic pathway or initiate the lectin pathway. The three complement activation pathways collectively cleave C3 into C3b and C3a, triggering terminal pathway activation, mainly involving C5-C9, which assembles to form the membrane attack complex (MAC). Key complement regulatory factors include C1 inhibitor (C1-INH), factor H (FH), factor I (FI), CD46, C4BP, and CD59. C1-INH inhibits C1r activation of C1s, preventing C4 and C2 cleavage. Simultaneously, C1-INH can inhibit the binding of MBL to MASP-1 or MASP-2. Factor I and factor H, aided by C4BP and CD46, can phagocytize C3 from the alternative pathway, inhibiting its activation. CD59 prevents C9 from binding to C5b678 to form MAC

The complement system, comprising over 50 diverse proteins and cleavage molecules such as proenzymes, proteases, anaphylatoxins, receptors, regulators, opsonins, multimolecular complexes, and pattern recognition molecules that provide host defense against foreign microbes or allografts, mediate inflammatory responses and maintain normal tissue homeostasis [3, 4]. The activation of complement relies on three precisely regulated activation systems: classical pathway (CL), alternative pathway (AP), and mannose-binding lectin (MBL) pathway (Fig. 1).

### Classical complement activation pathway

The classical pathway of complement activation begins with the C1 complex comprising C1q, C1r, and C1s subcomponent proteins. Initially, the spherical head of C1q recognizes IgG/IgM antigen–antibody complexes, causing the rearrangement and activation of C1r, followed by the activation of C1s within the C1r-C1s tetramer. Active C1s cleaves C4 into C4a and C4b, and subsequently, C4b binds to C2, which is cleaved by C1s into C2a and C2b. This forms the C4b2b complex, known as the classical pathway C3 convertase [5]. This convertase catalyzes the conversion of C3 to C3b and C3a. C3b molecules are deposited together, and the substrate specificity is switched to form the classical pathway C5 convertase (C3bBb3b and C3b4b2b). C5 convertase (C3bBb3b and C3b4b2b) has a high level of complement C5 affinity, which allows C5 to be dissolved and activated quickly [6, 7]. C5, the first complement factor formed by the membrane attack complex (MAC). After being lysed, C5 initiates the complex assembly that can be inserted into the target cell membrane. The assembly is formed around C5b and includes four complements—C6, C7, C8 and C9 [8].

The classical complement activation pathway is a crucial arm of the immune response, providing a rapid and potent means of neutralizing pathogens marked by antibodies.

### Alternative complement pathway

The alternative complement pathway operates independently of antibodies and involves only two main components: factors B and D [9]. Factor D, an active serine protease, binds to and activates Factor B [10]. The activated Factor B then joins with C3b(H2O) to create the C3 convertase C3(H2O)Bb [11]. This convertase triggers a positive feedback loop by cleaving natural C3 molecules into C3a and C3b. Additionally, C3b binding to C3 convertase forms the C5 convertase, which cleaves C5 into biologically active fragments, C5a and C5b [12]. C5b then recruits complement components C6, C7, C8, and C9 to form the membrane attack complex (MAC). The MAC inserts into the pathogen membrane, leading to cell lysis and destruction [13].

The alternative complement pathway offers rapid and innate protection against pathogens by identifying and flagging foreign surfaces for removal. Its ongoing, low-level activity plays a crucial role in immune surveillance, greatly enhancing the effectiveness of the complement system.

### Lectin pathway

The lectin pathway of the innate immune system is activated by recognizing specific carbohydrate patterns on pathogens. This activation involves pattern recognition molecules such as MBL, ficolins, or collectins binding to the pathogen’s surface, initiating a cascade that includes serine protease zymogens like MASP-1, MASP-2, MASP-3, and the nonenzymatic protein MAp19, where MASP-1 and MASP-2 serving as key enzymes [14]. Upon binding to the carbohydrate ligand, the MBL-MASP complex converts MASP from a zymogen to its activated form [15]. Subsequently, MASP generates C3 convertase (C4b2a), resulting in a reaction akin to the classical pathway.

The lectin pathway aids in opsonizing pathogens for recognition and phagocytosis by immune cells. Moreover, it initiates the complement cascade, resulting in the formation of the MAC for the destruction of targeted pathogens. It serves as a vital frontline defense in the immune response against infections.

## Complement regulatory proteins

The complement regulatory system, comprising soluble and membrane-bound factors, plays a crucial role in all three complement activation pathways. These regulators engage with specific complement components to intricately balance activation and inhibition, ensuring precise homeostasis. This balance safeguards our tissues, effectively preventing damage from foreign microorganisms.

### C1 inhibitor (C1-INH)

C1-INH is a key inhibitor that regulates both the complement system and coagulation cascade. It plays a key role in controlling the classical complement pathway by inhibiting activated C1s and C1r, as well as targeting factors XIIa, kallikrein, and factor XIa in the coagulation contact system [16]. By forming a covalent complex with C1s, it prevents its activation [17]. Additionally, it hinders the activation of MASP-1 and MASP-2, inhibiting the lectin pathway [18], and interferes with the interaction between C3b and factor B, preventing alternative pathway activation. This multifaceted activity underscores the critical role of C1-INH in maintaining immune balance and preventing harmful responses [19].

### FI

Factor I (FI) is a crucial serine protease in the complement system, crucial for regulating complement activation. Through enzymatic cleavage, FI indirectly inhibits the complement system by hindering the activation of complement factors C4b and C3b [20]. Working in coordination with other complement regulatory proteins, FI cleaves C3b and C4b into their inactive forms, preventing excessive complement cascade amplification and maintaining a balance in immune responses [21]. This regulatory function is essential for protecting host cells and tissues from damage caused by excessive or inappropriate complement activation.

### FH

Factor H (FH) is a vital complement regulatory protein that inhibits the alternative pathway of the complement system. FH accelerates the decay of C3 convertases (C3b, Bb) of the alternative pathway and acts as a cofactor for factor I-mediated C3b cleavage and inactivation [22]. Additionally, FH directly inhibits the formation of the C3bBC3bC3b complex by interacting with C3b, properdin (factor P), and FB in the presence of FI [23]. The regulatory actions of FH are crucial for preventing excessive and inappropriate complement activation, safeguarding host cells and tissues.

### C4BP

C4b-binding protein (C4BP) is a crucial regulator of the complement system, controlling the activation cascade by interacting with C4b. It serves as a cofactor for FI-mediated cleavage of C4b, leading to the formation of inactive C4b fragments [24]. By facilitating C4b degradation, C4BP inhibits the classical and lectin pathways, preventing excessive complement activation. Moreover, it disrupts the assembly of the C3 convertase (C4b2a) in the classical pathway, further regulating the complement cascade [25]. This pivotal regulatory role maintains immune balance and protects host cells and tissues from damage.

### CD46

CD46, also known as membrane cofactor protein (MCP), is pivotal in regulating complement activation by promoting the proteolysis and activation of FI. This activity results in the degradation of C3b and C4b, preventing the formation and amplification of C3 and C5 convertases, and ultimately inhibiting the downstream steps of the complement cascade [26]. The regulatory function of CD46 is crucial for maintaining immune homeostasis and preventing autologous cell lysis. By controlling complement activation on host cell surfaces, CD46 helps prevent inappropriate immune responses and protects cells from damage caused by uncontrolled complement activation [27, 28].

### CD59

CD59, also known as protection, plays a pivotal role in immune regulation by inhibiting the terminal complement pathway. It prevents MAC assembly by preventing C8 and C9 from joining the MAC complex [29]. This protective action ensures the preservation of host cells, preventing cell lysis and maintaining cellular integrity [30]. CD59's function as a terminal pathway inhibitor is indispensable for averting unintended cell damage and upholding immune homeostasis.

## Complement and transplantation

In transplantation, the complement system serves a dual role—acting as a protective mechanism against foreign tissues while also posing a potential risk for transplant-related complications [31]. Its importance is seen in graft rejection and incompatibility. When foreign tissues are transplanted, the complement system can activate through foreign tissue pathways. Studies indicate that xenotransplant rejection is primarily mediated by the classical and alternative pathways of complement, with no significant role played by the lectin (MBL) pathway [32]. Complement proteins C3a and C5a, along with the MAC activated by these pathways, play a crucial role in lysing xenografts.

Complement activation, especially when the recipient’s blood reacts against the transplanted organ, plays a key role in rejection [33]. Poor outcomes in clinical islet transplantation may be attributed to the occurrence of a destructive instant blood-mediated inflammatory response (IBMIR) [34]. Complement activation is a vital component of IBMIR, triggered after a thrombotic reaction. During this phase, pancreatic islets exposed to blood in the portal vein undergo a direct assault by the complement system [35, 36], primarily due to the extensive binding of antibodies against C4 and C3 on the surface of transplanted pancreatic islet cells [37].

Hyperacute allograft rejection (HAR) and ischemia–reperfusion injury (IRI) in xenografts are central factors contributing to heterogeneous transplant failures, and the complement system plays a pivotal role in both HAR and IRI. HAR, characterized by rapid rejection within 48 h post-transplantation, results from preexisting cytotoxic antibodies in the recipient binding to graft antigens, leading to severe complement-dependent rejection [38, 39]. IRI is frequently encountered in kidney transplantation, often attributed to blood flow disorders. The complement component C3 plays a pivotal role in inflammatory processes, with its elevation worsening IRI-induced acute kidney injury and stimulating the production of secondary epithelial cell chemokines, thereby contributing to local inflammation [40, 41].

Understanding and managing complement activation in transplantation is vital for enhancing success rates and the long-term functionality of transplanted organs. Therapeutic complement inhibitors effectively protect organs from inflammatory damages [42], and ongoing research is exploring genetic engineering strategies in donor pigs with human complement regulatory proteins (hCRP) to minimize the impact of complement system activation on xenograft survival [43]. These studies aim to develop new complement inhibition and immunomodulation strategies, enhancing transplantation outcomes.

## Gene-edited pigs

Pigs are considered excellent candidates for xenotransplantation due to their genetic, physiological, metabolic, and anatomical similarities to humans. They can be easily bred and raised in controlled environments, providing organs of suitable size for human transplantation [44, 45]. However, molecular incompatibility between pig donors and human recipients often leads to immune complications and xenotransplant rejection [46]. Technologies such as zinc finger nucleases [47], transcription activator-like effector (TALE) nucleases [48], and CRISPR/Cas [49–51] have enabled the efficient editing of pig genomes.

### Deleting xenoreactivity antigens

**Fig. 2:**
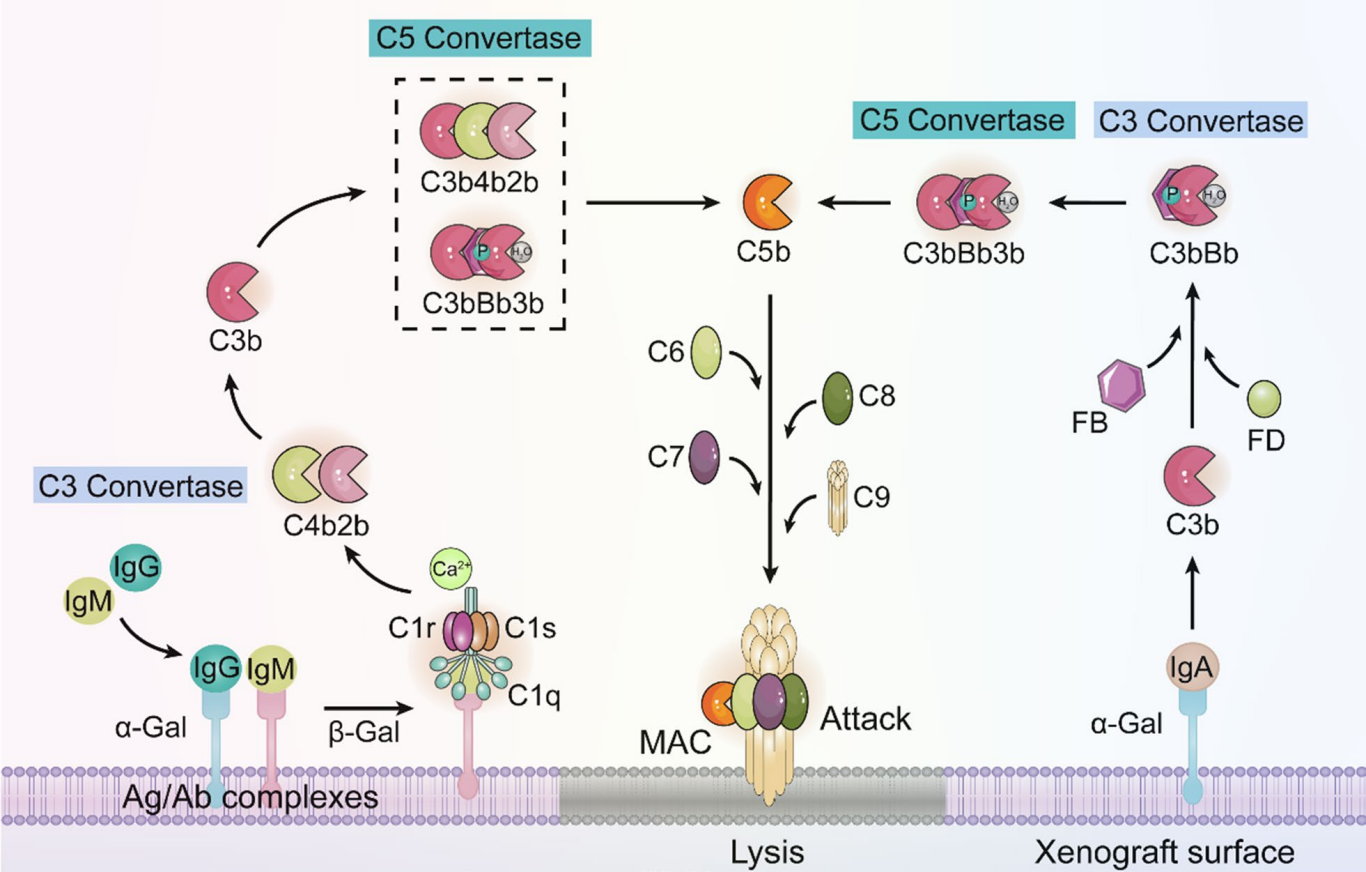
Xenograft activates the complement system. The binding of IgG/IgM to the protein α-Gal on the graft surface activates the classical pathway of complement, while the interaction of IgA with α-Gal activates the alternative pathway. The combined activation of classical and alternative pathways leads to the generation of C5 convertase, ultimately resulting in the formation of the membrane attack complex (MAC) composed of C5b, C6, C7, C8, and C9. This MAC complex functions to attack the xenograft

In pig-to-primate xenotransplantation, a major challenge is hyperacute rejection (HAR) occurring shortly after transplantation [52]. HAR is primarily due to preexisting antibodies in human plasma targeting the Gala (1,3)-Gal antigen on porcine endothelial cells (Fig. 2). This antigen is absent in humans and higher primates. Over 80% of complement-fixing xenoreactivity antibodies in human serum recognize Gala epitopes [53]. When xenoreactivity antibodies recognize and bind to Gala (1,3)-Gal antigen, the classical complement pathway is activated, leading to HAR induction. Therefore, preventing HAR requires strategies to neutralize the impact of anti-Gal antibodies or complement.

To make pig organs suitable for xenotransplantation, it is essential to eliminate the Gala (1,3) Gal antigen from xenograft cell surfaces. One approach involves deactivating the GGTA1 gene, responsible for forming the Gala (1,3) Gal epitope. In a study by Lai et al. [54], nuclear transfer successfully knocked out α-1,3-galactosyltransferase, producing pigs with heterozygous GGTA1 inactivation. Co-expressing human α1,2-fucosyltransferase and α-galactosidase significantly reduced Gal antigen levels on cell surfaces, thereby decreasing xenotransplant immunogenicity [55] (Fig. 2). Heterotopic heart transplantation from α-1,3-galactosyltransferase knockout pigs into baboons eliminated the galactose-α-1,3-galactose epitope, preventing HAR and extending porcine heart survival in baboons for 2–6 months. Additionally, homozygous α-1,3-galactosyltransferase knockout pigs were produced through breeding and somatic cell nuclear transfer (SCNT) [56].

### Expression of human complement regulatory proteins

To mitigate complement-mediated graft injury in xenotransplantation, genetically modifying pigs to express human complement regulatory proteins (hCRPs) like CD46 (membrane cofactor protein, MCP), CD55 (decay accelerating factor, DAF), and CD59 (membrane inhibitor of reactive lysis, MIRL) is an effective strategy [57, 58]. These hCRPs play a crucial role in maintaining the balance between complement activation and inhibition, acting as inhibitors at all stages of complement activation. The creation of multitransgenic pigs expressing CD46, CD55, and/or CD59 indicates that simultaneous expression of multiple hCRPs enhances protection [59]. Successful kidney transplantation from pigs expressing both hCD55 and hCD59 into nonimmunosuppressed baboons demonstrated their protective effect against hyperacute rejection (HAR) without immunosuppression [60].

Transplantation GTKO porcine hearts and kidneys significantly extend transplant survival [61, 62]. Compared to GTKO or CRP alone, incorporating hCRPs into GTKO pigs further reduces antibody-mediated rejections [63, 64]. Human CD55 expression effectively blocks HAR and limits local complement activation in GTKO heart transplantation [64]. Additionally, GTKO combined with human CD46 transgenic (GKO/CD46) islets enhances xenograft survival by mitigating early platelet deposition and neutrophil infiltration [65]. These findings collectively suggest that GGTA1 knockout pigs with one or two hCRPs are more suitable donors for organ xenotransplantation.

## Gene-edited pig organ transplantation model

**Fig. 3:**
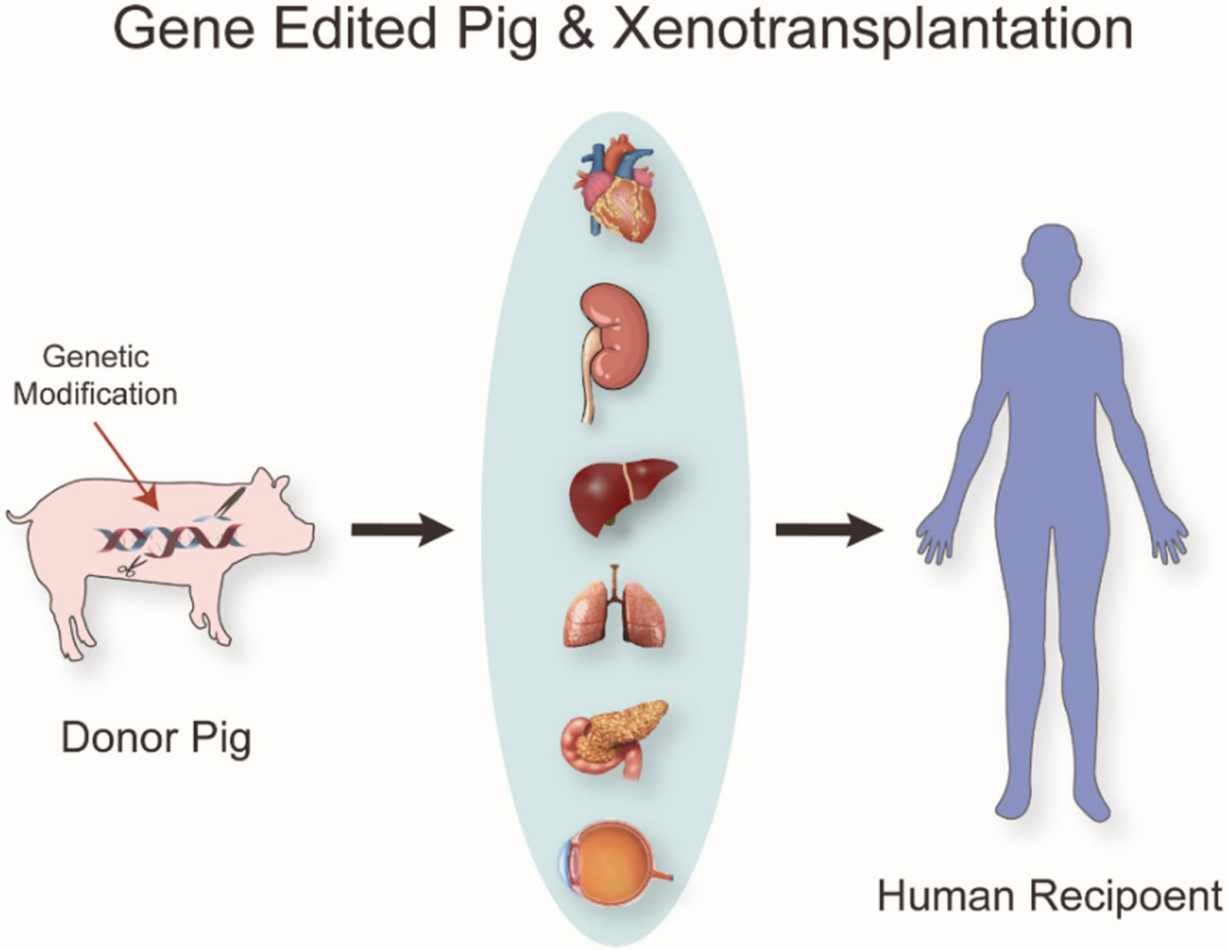
Gene-edited pig & xenotransplantation. Organs cultivated from genetically modified cloned pigs, such as heart, liver, lung, kidney, etc., can be transplanted into patients

Ongoing advancements in gene editing technology, paired with continual enhancements in immunosuppressive regimens, have significantly propelled the evolution of pig-to-non-human primate (NHP) organ transplant models. Pigs now serve as vital organ donors in the field of xenotransplantation (Fig. 3).

### Heart transplant

Most porcine heart transplant studies involve heterotopic transplantation [66, 67]. Until 2005, the longest median survival time for porcine hearts transplanted heterotopically into baboons was 96 days [68]. With an immunosuppressive regimen comprising αTG, anti-CD154, and MMF, effective B cell depletion with the anti-CD20 antibody extended the survival time of heterotopic cardiac transplants to 236 days. However, delayed rejection eventually led to graft failure [69]. In 2016, the same researchers utilized GTKO/hCD46/hTBM donor porcine hearts for transplantation into baboons. Implementing an αCD40 antibody-based immunomodulatory regimen (2C10R4), the longest survival time for heterotopic heart transplantation was extended to an impressive 945 days, with a median survival time of 298 days [70]. Non-human primate studies show that both ectopic and orthotopic heart transplants maintain function and prolong survival. This is attributed to sustained cardioplegic solutions during hypothermic ischemia and an effective immunosuppressive regimen [71]. These preclinical findings pave the way for successful xenotransplantation of genetically modified porcine hearts into patients with end-stage heart failure.

In recent years, several international teams have initiated preclinical studies on gene-edited pig heart transplantation. On January 7, 2022, surgeons at the University of Maryland successfully performed the world's first transgenic pig heart transplant on a 57-year-old man. Despite the patient's death 60 days after surgery, this procedure marked a groundbreaking moment in xenotransplantation history [72]. Notably, it overcame potential postoperative obstacles such as hyperacute immune rejection, achieved favorable short-term outcomes, and emphasized the necessity for further clinical research. However, viral safety was overlooked during this xenotransplantation process, leading to the transmission of pig viruses (PCMV/PRV) to human recipients, resulting in patient fatalities [73]. In July 2023, Nader Moazami et al. transplanted hearts from 10 gene-edited pigs into two brain-dead human recipients and meticulously monitored transplant function, pre-existing xenoreactivity antibody injury, hemodynamics, and systemic reactions within 66 h. They found no safety risks in the deceased recipients [74]. While progress has been made, it’s clear we're not fully prepared for human xenotransplantation.

### Kidney transplant

The utilization of kidneys in pig-to-NHP models has progressed more slowly compared to heart transplants. Wild-type pig kidneys typically fail in NHPs within a few minutes [75]. In 2004, the transplantation of CD55 pig kidneys into a cynomolgus monkey model revealed that the average survival of pig renal xenotransplants was limited to several weeks, with 90 days being the longest reported survival in a pig-to-NHP model [76].

Until Higginbotham et al.'s breakthrough in 2015, progress in pig xenotransplantation had been limited. In their study, GGTA1 KO/hCD55 pig kidneys were transplanted into rhesus monkeys. After T cell exhaustion, the treatment was sustained by stimuli blocking in addition to the maintenance of mycophenolate mofetil and steroids. Remarkably, the graft survived for an impressive 310 days [77]. Subsequent experiments with gene-edited pigs have shown promising results. Iwase et al. conducted kidney transplants from GTKO/CD46/CD55/TBM/EPCR/CD39 pigs to baboons treated with an anti-CD40mAb-based regimen, with kidney function enduring for 136 days [78]. Kim et al. achieved the longest reported life-sustaining xenotransplantation, lasting 499 days, by transplanting GGTA1KO/hCD55 pig kidneys into rhesus monkeys with low anti-pig antibody titers and selective depletion of CD4 + or CD8 + T cells, emphasizing the significance of CD4 + T cell depletion [79].

Before the first global porcine heart transplantation to humans, three instances of genetically modified pig kidney transplants into humans occurred. Two recipients received genetically modified GTKO porcine kidneys, maintaining normal renal functions for 54 h without signs of HAR or antibody-mediated rejection [80]. In the third case, the donor kidneys (bilateral transplantation) had ten genetic modifications, and three pig carbohydrate antigens and the pig growth hormone receptor gene were deleted. These gene-modified kidneys functioned for 74 h without rejection or antibody or complement protein deposition [81].

In summary, the added expression of several hCRPs in GTKO transgenic pigs can further prevent rejection development [82]. With continuous scientific efforts, life-sustaining kidney xenotransplantation is much closer to clinical reality than previously thought.

### Liver transplant

Liver xenotransplantation from pigs to humans faces considerable hurdles compared to heart and kidney transplants, including immunological complexities, clotting abnormalities, and rejection risks. Ekser et al. pioneered the transplantation of GTKO or GTKO/hCD46 pig livers into baboons, resulting in severe thrombocytopenia with a survival of 7 days [83, 84]. In 2012, Kim transplanted GTKO livers into baboons and achieved up to 9 days of porcine liver xenotransplantation under the same immunosuppression regimen as xenotransplantation of heart and kidney [85]. The Shah team explored the effects of exogenous administration of human coagulation factors after pig-to-baboon liver xenotransplantation (LXT) using GTKO pig donors. The addition of costimulatory blockade to this regimen increased individual recipients’ LXT survival from 9 to 25 days [86, 87]. In their modified experimental protocol, costimulation blockade (belatacept or anti-CD40mAb) extended the 25 day survival period to 29 days [88]. The outlook for pig-to-NHP liver transplantation depends on ongoing advancements in genetic engineering, immunosuppressive protocols, and a deeper understanding of the immunological and physiological factors involved in xenotransplantation.

### Lung transplant

Xenotransplantation faces heightened challenges in lung transplantation due to the lung's sensitivity to injury and multiple immune rejection mechanisms. During transplantation, the pig lung is the organ most severely damaged due to rapid coagulation dysfunction [89]. While progress has been made in heart and kidney transplantation with GTKO and hCD46, lung xenografts still face challenges. In 2007, Nguyen et al. transplanted GTKO left lungs into baboons, but these lungs could only sustain life for 3.5 h due to severe coagulation disorders [90]. Laird et al. discovered that transgenic expression of human leukocyte antigen-E attenuated GTKO/hCD46 pig lung xenograft injury, prolonging survival *ex vivo* [91]. Watanabe et al. proposed that transgene expression of hCD47 on porcine blood vessels could alleviate acute vascular rejection in baboons, particularly in GTKO pig lungs that are highly susceptible [92]. Utilizing multigene donor pigs, combined with targeted complement activation (hCD46, hCD55), coagulation (hEPCR, hVWF, hTBM, hTFPI, hCD39), and anti-inflammatory pathway regulatory genes (HO-1, HLA-E), significantly improved the survival of xenogeneic swine lungs in both ex vivo human blood perfusion and in life-supporting in vivo models [93]. However, the survival rate of lung xenotransplantation remains measured in days rather than weeks or months [94].

### Islet transplantation

The primary challenges in the early stages of porcine islet transplantation are HAR and IBMIR [95]. When the NHP immune system detects Galα (1,3) in porcine tissue, it triggers the classical complement pathway, resulting in the formation of membrane attack complexes and cell lysis. Thus, knockdown of the islet surface α-Gal epitope is therefore a logical choice [96]. In addition to removing the α-Gal antigen, reducing IBMIR also requires the expression of human complement regulatory factors (hCD46, hCD55, hCD59) [97]. When porcine pancreatic islets are transplanted into non-human primates (NHPs) with diabetes, a significant portion of the graft is typically lost early due to IBMIR and intense immunosuppressive therapy [98]. Windt et al. addressed complement activation by expressing hCD46 on pig islets, effectively limiting antibody-mediated rejection and preserving islet quality. While this approach didn’t prevent the immediate loss of most transplanted islets, it significantly improved the outcomes of islet xenotransplantation in diabetic cynomolgus monkeys, maintaining normoglycemia for over 12 months [99, 100]. Hawthorne et al. achieved minimal IBMIR when transplanting α-Gal-deficient pigs with hCD55 and hCD59 transgenes onto neonatal islet cell clusters, combined with a clinically relevant immunosuppressive protocol [101]. Achieving clinical long-term survival of pancreatic islets may require more effective immunosuppression or further modification of donor genes.

### Corneal transplantation

Porcine corneal xenotransplantation is considered feasible due to the cornea’s immune privilege and avascular nature [102]. Recent nonhuman primate studies have shown promising results, indicating that porcine xenografts can endure for an extended period during corneal transplantation [103]. Dong et al. discovered that corneas from GTKO/CD46 pigs did not significantly improve graft survival compared to those from wild-type pigs. Prolonging the survival of corneal xenografts in pig-to-monkey corneal xenotransplantation encounters challenges in preventing anterior synechiae and retrocorneal membrane formation [104]. The use of decellularized corneas from wild-type pigs for anterior lamellar keratoplasty has shown graft transparency for over a year [105]. In treating corneal fungal ulcers and other clinical diseases, Zhang et al. reported that implantation of acellular porcine corneal stromata (APCS) resulted in no recurrence of infection during a 6 month follow-up period, and all corneal ulcers healed with neovascularization. APCS grafts proved safe and efficacious in lamellar keratoplasty for various clinical conditions [106]. As genetically engineered pigs become more available, pig corneas have the potential to address the global shortage of corneas soon.

## Research and application of complement-related drugs

Advances in comprehending the complement system's composition, structure, and interactions have paved the way for developing drugs with both stimulating and inhibitory effects on complement activity. These medications show potential as therapies for a wide range of conditions, including infectious, inflammatory, traumatic, cancerous, autoimmune, or age-related diseases, as well as for preventing transplant rejection.

Four C1-INH drugs have been approved [107]: Cinryze, Berinert, Ruconest, and Cetor. C1-INH inhibits the kinin B1 receptor, reduces the release of chemotactic microvesicles from damaged donor tissues, and effectively prevents late antibody-mediated rejection. C1-INH has a longstanding history of treating hereditary angioedema (HAE) with high safety and effectiveness [108]. While data on its use in organ transplantation are limited, experimental evidence suggests potential benefits in alleviating acute antibody-mediated rejection (ABMR) observed in baboons during transplantation [109]. A case report on Cinryze's use in treating acute ABMR post-kidney transplantation indicates promising outcomes [110]. Berinert, according to data from a phase 1/2 study involving 20 patients, may enhance allograft function in kidney recipients with unresponsive acute ABMR [111]. However, there is still limited research on Ruconest and Cetor in organ transplantation.

Inhibiting the complement system at the C3 level effectively prevents unregulated activation, safeguarding host cells from damage. Cobra venom factor (CVF), an analog of complement component C3, exhibits specific anti-complement C3 biological activity. CVF induces lysis of C3 and C5, depleting complement C3 (and C5), inhibiting humoral immunity, and has found widespread use in xenotransplantation due to its long-term complement removal effects [112, 113]. Cp40, an analog of compstatin, is a potent inhibitor of complement C3. It effectively prevents C3 activation and mitigates complement-mediated injury triggered by endothelial antibody binding and extracorporeal circulation [114]. Cp40 demonstrates the ability to inhibit complement activation, promote anti-inflammatory and anticoagulant effects in septic animals [115]. Notably, Cp40 can prevent the adhesion of leukocytes, specifically neutrophils, to porcine endothelium [116]. Considering these findings, Cp40 stands out as a promising adjunct for preclinical and future clinical cardiac xenotransplantation [117].

Several interventions are currently being explored to prevent xenograft injury and improve its survival rate. Eculizumab, a recombinant antibody targeting complement C5, holds the potential for reducing antibody-mediated rejection [118]. As the first anti-complement drug, eculizumab offers a novel therapeutic approach for various human diseases, reshaping treatment strategies for conditions like PNH and significantly impacting their clinical outcomes [119, 120].

**Fig. 4:**
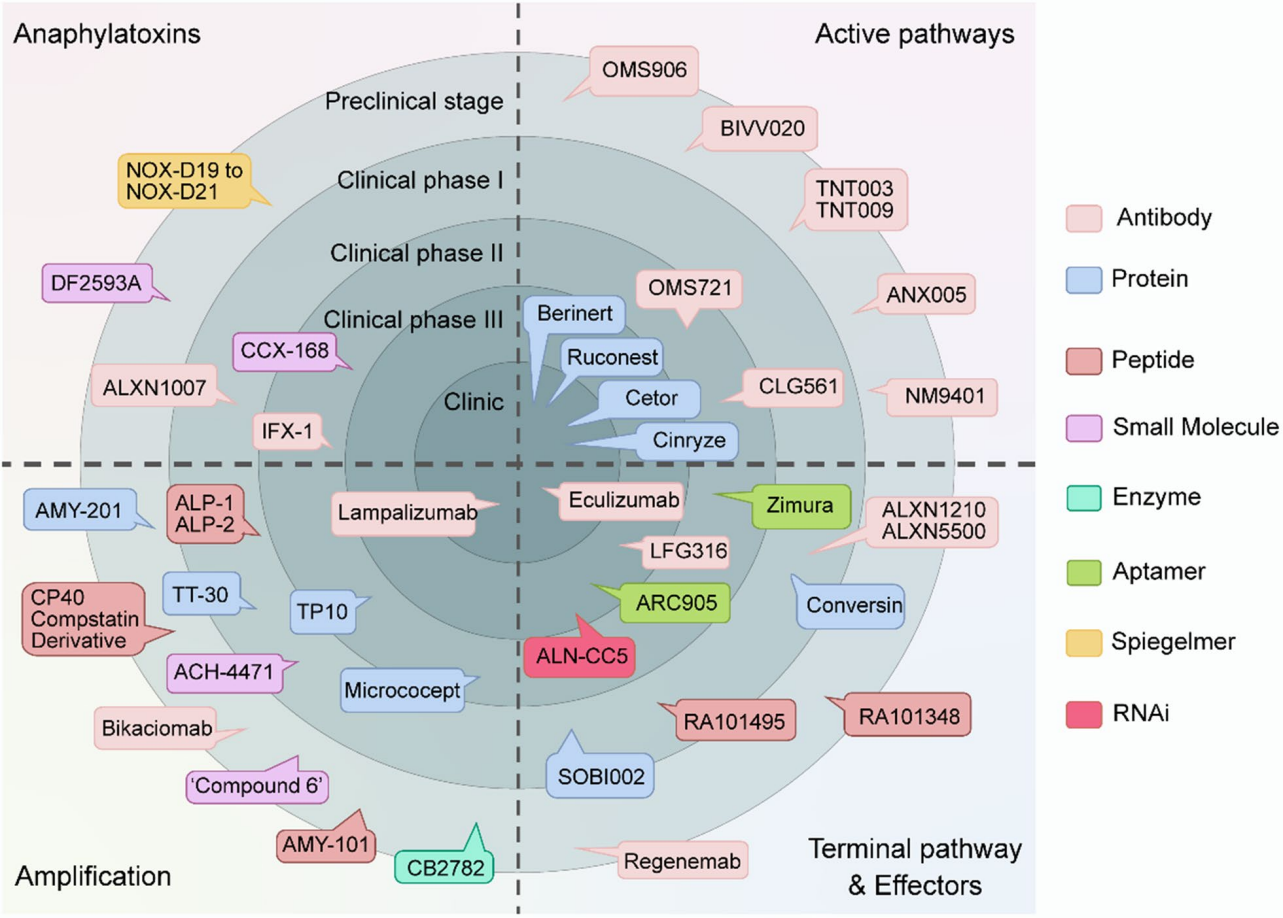
Preclinical and clinical trials of complement therapy. Complement therapy progresses through different stages, spanning from preclinical work to market authorization. These stages include laboratory research, animal models, clinical phases I, II, and III, and final clinical implementation. The therapeutic goals are categorized into four quadrants, representing major complement categories: anaphylatoxins, active pathways, amplification and terminal pathways, and effectors. Each arrow denotes a specific agent and its development stage. Drugs targeting C1r/s and MASP include Cinryze (Shire), Berinert (CSL Behring), Cetor (Sanquin), and Ruconest (Pharming), which are already being used in clinics; drugs targeting C1q include ANX005 (Annexon); drugs targeting C1s include TNT003 (True North), TNT009 (True North), and BIVV020 (Sanofi); TP10 (CDX-1135; Celldex Therapeutics) targets the soluble form of complement receptor type 1 (CR1); OMS721 (Narsoplimab, Omeros) targets the MASP-2 target; Drugs targeting MASP-3 include OMS906 (Omeros); drugs targeting Properdin include CLG561 (Novartis) and NM9401 (Novelmed); drugs targeting C3 include AMY-101 (Amyndas), APL-1 (Apellis), APL-2 (Apellis), CB2782 (Catalyst), Cp40 (Amyndas); drugs targeting C3b and convertases include AMY-201 (Amyndas), and Mirococept (MRC); drugs targeting FB include Bikaciomab (Novelmed); drugs targeting FD include Lampalizumab (Genentech), ACH-4471 (Achillion), and “Compound 6”(Novartis); drugs targeting C5 include Eculizumab (Soliris, Alexion), ALXN1210 (Alexion), ALXN5500 (Alexion), LFG316 (Novartis), Coversin (Akari), RA101495 (Ra Pharma), ALN-CC5 (Alnylam), RA101348 (RaPharma), ARC 1905 (Zimura; Ophthotech), and the affibody SOBI002 (Swedish, Orphan Biovitrum) targets C5 (programme recently terminated); drugs targeting C5a include IFX-1 (InflaRx), ALXN-1007 (Alexion), NOX-D21 (Noxxon Pharma); drugs targeting C5aR include CCX168 (Chemocentryx); C6 target drug includes Regenemab (Regenesance); CR2–FH target drug includes TT30 (ALXN 1102; Alexion)

Identifying suitable complement inhibitors and defining therapeutic strategies is crucial for future studies. Genetically engineering pigs with appropriate human complement modulators emerges as a promising strategy in xenotransplantation [121]. Figure 4 outlines development programs focusing on inhibitors against various complement targets, with some undergoing clinical studies in both healthy individuals and patients [122–125].

## Conclusions and perspective

The global shortage of organs for transplantation has led to the investigation of xenotransplantation as a potential solution. This method, which involves transplanting organs from genetically modified pigs into humans, offers hope in alleviating the scarcity of human organ donors. Research indicates that graft failure often stems from the activation of the complement system, affecting critical aspects of xenografts, including galactosidase binding, antibody interactions, and complex responses involving coagulation, inflammation, and adaptive immune reactions during transplantation.

The recent breakthroughs in gene-edited porcine heart transplantation represent significant progress and offer valuable insights for refining this approach [73]. However, the presence of pig viruses in gene-edited pig xenotransplantation cannot be overlooked [126]. Despite this, these successes not only shed light on the long-term viability of cardiac xenotransplants but also lay a foundation for transplanting other organs into humans. The lessons learned from porcine heart transplantation serve as a crucial reference for optimizing xenotransplantation, which involves strategically using genetically modified organs to evade the human immune response. The implementation of advanced gene-editing techniques, including CRISPR/Cas9, TALEN, and SCNT, in modifying potential pig donors has led to substantial advancements in xenotransplantation. Genetically engineered pig organs, combined with novel immunosuppressive therapies, have extended the survival rates in NHP xenotransplants.

The ongoing development of complement-related clinical drug candidates provides a diverse array of options for selective inhibition, targeting, and drug delivery, contributing to the progress of xenotransplantation. Despite facing immunobiological challenges, the increasing variety of genetically modified pigs and the expansion of immunosuppressant and anti-inflammatory drugs offer optimism. Clinical trials for pig kidney, heart, liver, lung, pancreatic islet, and corneal transplantation are anticipated, bringing animal organ transplantation into reality for human recipients shortly.

## Data Availability

Not applicable.

## Abbreviations

ABMR: Antibody-mediated rejection
AP: Alternative pathway
APCS: Acellular porcine corneal stromata
C1-INH: C1 inhibitor
C4BP: C4b-binding protein
CL: Classical pathway
CVF: Cobra venom factor
EPCR: Endothelial protein C receptor
FI: Factor I
FH: Factor H
GHR: Growth hormone receptor
HAE: Hereditary angioedema
HAR: Hyperacute allograft rejection
hCRP: Human complement regulatory protein
hHO-1: Heme oxygenase-1
HLA: Human leukocyte antigen
IBMIR: Instant blood-mediated inflammatory response
IRI: Ischemia–reperfusion injury
LXT: Liver xenotransplantation
MAC: Membrane attack complex
MAP: Membrane attack protein
MBL: Mannose binding lectin
MCP: Membrane cofactor protein
MIRL: Membrane inhibitor of reactive cleavage
NHP: Non-human primate
PERVs: Porcine endogenous retroviruses
SCNT: Somatic cell nuclear transfer
TALE: Transcription activator-like effector

## References

[1] <https://www.organdonor.gov/learn/organ-donation-statistics> (Health Resources & Services Administration).

[2] Rao JS, Matson AW, Taylor RT, Burlak C (2021). Xenotransplantation literature update january/february 2021. Xenotransplantation.

[3] Bohlson SS, Garred P, Kemper C, Tenner AJ (2019). Complement nomenclature-deconvoluted. Front Immunol.

[4] Vonbrunn E, Büttner-Herold M, Amann K, Daniel C (2023). Complement inhibition in kidney transplantation: where are we now?. BioDrugs.

[5] Daha R, VK C,  Roos A (2006). Compliments from complement: a fourth pathway of complement activation. Nephrol Dial Transplant.

[6] Mannes M (2021). Complement inhibition at the level of C3 or C5: mechanistic reasons for ongoing terminal pathway activity. Blood.

[7] Rawal N, P M (2003). Formation of high-affinity C5 convertase of the classical pathway of complement. J Biol Chem.

[8] Pangburn MK, Ra N (2002). Structure and function of complement C5 convertase enzymes. Biochem Soc Trans.

[9] Lenderink AM, L K, Ljubanović D, Coleman KE (2007). The alternative pathway of complement is activated in the glomeruli and tubulointerstitium of mice with adriamycin nephropathy. Am J Physiol Renal Physiol.

[10] Angela Martin PJ, Lachmann LH, Hobart MJ (1976). Haemolytic diffusion plate assays for factors B and D of the alternative pathway of complement activation. Immunochemistry.

[11] Dodds AW, Smith SL, Paul Levine R, Willis AC (1998). Isolation and initial charac terisation of complement components C3 and C4 of the nurse shark and the channel catfish. Immunology.

[12] Uber-Lang M, S J, Zetoune FS, Rittirsch D, Neff TA, McGuire SR, Lambris JD, Warner RL, Flierl MA, Hoesel LM (2006). Generation of C5a in the absence of C3: a new complement activation pathway. Nat Med.

[13] Doryen B (2014). The making of a macromolecular machine: assembly of the membrane attack complex. Biochemistry.

[14] Thiel S, V-J T, Stover CM, Schwaeble W (1997). A second serine protease associated with mannan-binding lectin that activates complement. Nature.

[15] Dahl MR, Thiel S, Matsushita M, Fujita T, Willis AC, Christensen T, Vorup-Jensen T, Jensenius JC (2007). its association with distinct complexes of the mannan-binding lectin complement activation pathway. Immunity.

[16] Davis AE, Mejia P, Fengxin Lu (2008). Biological activities of C1 inhibitor. Mol Immunol.

[17] Wouters D, Wagenaar-Bos I, van Ham M (2008). C1 inhibitor: just a serine protease inhibitor? New and old considerations on therapeutic applications of C1 inhibitor. Expert Opin Biol Ther.

[18] Mauriello CT, Pallera HK, Sharp JA, Woltmann JL, Qian S, Hair PS (2013). A novel peptide inhibitor of classical and lectin complement activation including ABO incompatibility. Mol Immuno.

[19] Bergamaschini L, Gobbo G, Gatti S, Caccamo L, Prato P, Maggioni M, Braidotti P, Di Stefano R, Fassati LR (2002). Endothelial targeting with C1-inhibitor reduces complement activation in vitro and during ex vivo reperfusion of pig liver. Clin Exp Immunol.

[20] Escudero-Esparza A, Kalchishkova N (2013). The novel complement inhibitor human CUB and Sushi multiple domains 1 (CSMD1) protein promotes factor I-mediated degradation of C4b and C3b and inhibits the membrane attack complex assembly. FASEB.

[21] Asakawa R, Komatsuzawa H, Kawai T, Yamada S, Goncalves RB (2003). Outer membrane protein 100, a versatile virulence factor of *Actinobacillus actinomycetemcomitans*. Mol Microbiol.

[22] Ferreira VP, Pangburn MK, Cortés C (2010). Complement control protein factor H: the good, the bad, and the inadequate. Mol Immunol.

[23] Whaley K, Ruddy S (1976). Modulation of the alternative complement pathways by beta 1 H globulin. J Exp Med.

[24] Scharfstein J, Ferreira A, Gigli I, Nussenzweig V (1978). Human C4-binding protein. I isolation and characterization. J Exp Med.

[25] Blom AM, Zadura AF, Villoutreix BO, Dahlba ¨ck B (2000). Positively charged amino acids at the interface between α-chain CCP1 and CCP2 of C4BP are required for regulation of the classical C3-convertase. Immunology.

[26] Nilsson SC, Sim RB, Lea SM, Fremeaux-Bacchi V, Blom AM (2011). Complement factor I in health and disease. Immunol Immunol.

[27] Rooijakkers SHM, van Strijp JAG (2007). Bacterial complement evasion. Immunol.

[28] Ipfel PF, Würzner R, Skerka C (2007). Complement evasion of pathogens: common strategies are shared by diverse organisms. Immunol.

[29] Meri S, PM B, Davies A, Daniels RH, Olavesen MG, Waldmann H, Lachmann PJ (1990). Human protectin (CD59), an 18000–20000 MW complement lysis restricting factor, inhibits C5b–8 catalysed insertion of C9 into lipid bilayers. Immunology.

[30] Rollins SA, Zhao J, Ninomiya H (1999). Inhibition of homologous complement by CD59 is mediated by a species-selective recognition conferred through binding to C8 within C5b–8 or C9 within C5b–9. J Immunol.

[31] Markiewski MM, Lambris JD (2007). The role of complement in inflammatory diseases from behind the scenes into the spotlight. Am J Pathol.

[32] Broom, C. U., M. E. Methods of treating antibody-mediated rejection in organ transplant patients with C1-esterase inhibitor. (2018).

[33] Biglarnia A-R, Huber-Lang M, Mohlin C, Ekdahl KN, Nilsson Bo (2018). The multifaceted role of complement in kidney transplantation. Nat Rev Nephrol.

[34] Akima S, Hawthorne WJ, Favaloro E (2009). Tirofiban and activated protein C synergistically inhibit the instant blood mediated inflammatory reaction (IBMIR) from allogeneic islet cells exposure to human blood. Am J Transplant.

[35] Berman DM, Cabrera O, Kenyon NM (2007). Interference with tissue factor prolongs intrahepatic islet allograft survival in a nonhuman primate marginal mass model. Transplantation.

[36] Bennet W, Sundberg B, Groth CG (1999). Incompatibility between human blood and isolated islets of langerhans: a finding with implications for clinical intraportal islet transplantation. Diabetes.

[37] Tjernberg J, Ekdahl KN, Lambris JD, Korsgren O (2008). Acute antibody-mediated complement activation mediates lysis of pancreatic islets cells and may cause tissue loss in clinical islet transplantation. Transplantation.

[38] Hyperacute rejection. *N Engl J Med* **279**, 657–658, doi:10.1056/nejm196809192791209 (1968). 10.1056/NEJM196809192791209 4875679

[39] Grafals M, Thurman JM (2019). The role of complement in organ transplantation. Front Immunol.

[40] Peng Q (2012). C3a and C5a promote renal ischemia-reperfusion injury. J Am Soc Nephrol.

[41] Thurman JM (2007). C3a is required for the production of CXC chemokines by tubular epithelial cells after renal ischemia/reperfusion. J Immunol.

[42] Vonbrunn E, Buttner-Herold M, Amann K, Daniel C (2023). Complement inhibition in kidney transplantation: where are we now?. BioDrugs.

[43] Butler JR, Ladowski JM, Martens GR, Tector M, Tector AJ (2015). Recent advances in genome editing and creation of genetically modified pigs. Int J Surg.

[44] Heng Z, Kaixiang X, Ninglin F, Hongye Z, Hongjiang W (2018). Construction and status quo of gene-edited xenotransplantation pigs. Electron J Pract Organ Transplant.

[45] Hryhorowicz M, Zeyland J, Słomski R, Lipiński D (2017). Genetically modified pigs as organ donors for xenotransplantation. Mol Biotechnol.

[46] Arabi TZ, Sabbah BN, Lerman A, Zhu XY, Lerman LO (2023). Xenotransplantation: current challenges and emerging solutions. Cell Transplant.

[47] Miller JC, Holmes MC, Wang J, Guschin DY, Lee Y-L, Rupniewski I, Beausejour CM, Waite AJ, Wang NS, Kim KA, Gregory PD, Pabo CO, Rebar EJ (2007). An improved zinc-finger nuclease architecture for highly specific genome editing. Nat Biotechnol.

[48] Schmid-Burgk JL, Schmidt T, Kaiser V, Höning K, Hornung V (2013). A ligation-independent cloning technique for high-throughput assembly of transcription activator-like effector genes. Nat Biotechnol.

[49] Hsu PD, Lander ES, Zhang F (2014). Development and applications of CRISPR-Cas9 for genome engineering. Cell.

[50] Yaoqiang H, Guoling L, Huaqiang Y, Zhenfang W (2018). Application of gene-edited pigs in biomedical research. Genetic.

[51] Tanihara F (2021). One-step generation of multiple gene-edited pigs by electroporation of the CRISPR/Cas9 system into zygotes to reduce xenoantigen biosynthesis. Int J Mol Sci.

[52] Deng J (2022). Advance of genetically modified pigs in xeno-transplantation. Front Cell Dev Biol.

[53] Jager U, Takeuchi Y, Porter C (1999). Induction of complement attack on human cells by Gal(alpha1,3)Gal xenoantigen expression as a gene therapy approach to cancer. Gene Ther.

[54] Lai L, Kolber-Simonds D, Park K-W, Cheong H-T, Greenstein JL, Im G-S, Samuel M, Bonk A, Rieke A, Day BN, Murphy CN, Carter DB, Hawley RJ, Prather RS (2002). Production of alpha-1,3-galactosyltransferase knockout pigs by nuclear transfer cloning. Science.

[55] Zeyland J, Woźniak A, Gawrońska B, Juzwa W, Jura J, Nowak A, Słomski R, Smorąg Z, Szalata M, Mazurek U, Lipiński D (2014). Double transgenic pigs with combined expression of human α1,2-fucosyltransferase and α-galactosidase designed to avoid hyperacute xenograft rejection. Arch Immunol Ther Exp.

[56] Nottle MB, Beebe LFS, Harrison SJ, McIlfatrick SM, Ashman RJ, O’Connell PJ, Salvaris EJ, Fisicaro N, Pommey S, Cowan PJ, D’Apice AJF (2007). Production of homozygous alpha-1,3-galactosyltransferase knockout pigs by breeding and somatic cell nuclear transfer. Xenotransplantation.

[57] Gelderman KA, Blok VT, Fleuren GJ, Gorter A (2002). The inhibitory effect of CD46, CD55, and CD59 on complement activation after immunotherapeutic treatment of cervical carcinoma cells with monoclonal antibodies or bispecific monoclonal antibodies. Lab Invest.

[58] Zhou C-Y, McInnes E, Copeman L, Langford G, Parsons N, Lancaster R, Richards A, Carrington C, Thompson S (2010). Transgenic pigs expressing human CD59, in combination with human membrane cofactor protein and human decay-accelerating factor. Xenotransplantation.

[59] Huang J, Gou D, Zhen C, Jiang D, Mao X, Li W, Chen S, Cai C (2001). Protection of xenogeneic cells from human complement-mediated lysis by the expression of human DAF, CD59, and MCP. FEMS Immunol Med Microbiol.

[60] Ménoret S, Plat M, Blancho G, Martinat-Botté F, Bernard P, Karam G, Tesson L, Renaudin K, Guillouet P, Weill B, Chéreau C, Houdebine L-M, Soulillou J-P, Terqui M, Anegon I (2004). Characterization of human CD55 and CD59 transgenic pigs and kidney xenotransplantation in the pig-to-baboon combination. Transplantation.

[61] Shimizu A, Hisashi Y, Kuwaki K, Tseng Y-L, Dor FJMF, Houser SL, Robson SC, Schuurman H-J, Cooper DKC, Sachs DH, Yamada K, Colvin RB (2008). Thrombotic microangiopathy associated with humoral rejection of cardiac xenografts from α1,3-galactosyltransferase gene-knockout pigs in baboons—sciencedirect. Am J Pathol.

[62] Hisashi Y, Yamada K, Kuwaki K, Tseng Y-L, Dor FJMF, Houser SL, Robson SC, Schuurman H-J, Cooper DKC, Sachs DH, Colvin RB, Shimizu A (2008). Rejection of cardiac xenografts transplanted from alpha1,3-galactosyltransferase gene-knockout (GalT-KO) pigs to baboons. Am J Transplant.

[63] Azimzadeh AM, Kelishadi SS, Ezzelarab MB, Singh AK, Stoddard T, Iwase H, Zhang T, Burdorf Lars, Sievert E, Avon C, Cheng X, Ayares D, Horvath Keith A, Corcoran PC, Mohiuddin MM, Barth RN, Cooper DKC, Pierson RN (2015). Early graft failure of GalTKO pig organs in baboons is reduced by expression of a human complement pathway-regulatory protein. Xenotransplantation.

[64] McGregor CGA, Ricci D, Miyagi N, Stalboerger PG, Zeji D, Oehler EA, Tazelaar HD, Byrne GW (2012). Human CD55 expression blocks hyperacute rejection and restricts complement activation in Gal knockout cardiac xenografts. Transplantation.

[65] Samy KP, Gao Q, Davis RP, Song M, Fitch ZW, Mulvihill MS, MacDonald AL, Leopardi FV, How T, Williams KD, Devi GR, Collins BH, Luo X, Kirk AD (2019). The role of human CD46 in early xenoislet engraftment in a dual transplant model. Xenotransplantation.

[66] Tianyu L, Yang B, Wang R, Qin C (2020). Xenotransplantation: current status in preclinical research. Front Immunol.

[67] Boulet J, Cunningham JW, Mehra MR (2022). Cardiac xenotransplantation: challenges, evolution, and advances. JACC Basic Trans Sci.

[68] McGregor CGA, Davies WR, Oi K, Teotia SS, Schirmer JM, Risdahl JM, Tazelaar HD, Kremers WK, Walker RC, Byrne GW, Logan JS (2005). Cardiac xenotransplantation: recent preclinical progress with 3-month median survival. J Thor Cardiovasc Surg.

[69] Mohiuddin MM, Corcoran PC, Singh AK, Azimzadeh A, Hoyt RF, Thomas ML, Eckhaus MA, Seavey C, Ayares D, Pierson RN, Horvath KA (2012). B-cell depletion extends the survival of GTKO.hCD46Tgpig heart xenografts in baboons for up to 8 months. Am J Transplant.

[70] Mohiuddin MM, Singh AK, Corcoran PC, Thomas III ML, Clark T, Lewis BG, Hoyt RF, Eckhaus M, Pierson III RN, Belli AJ, Wolf E, Klymiuk N, Phelps C, Reimann KA, Ayares D, Horvath KA (2016). Chimeric 2C10R4 anti-CD40 antibody therapy is critical for long-term survival of GTKO.hCD46.hTBM pig-to-primate cardiac xenograft. Nat Commun.

[71] Garry DJ, Weiner JI, Greising SM, Garry MG, Sachs DH (2022). Mechanisms and strategies to promote cardiac xenotransplantation. J Mol Cell Cardiol.

[72] Mohiuddin MM (2023). Graft dysfunction in compassionate use of genetically engineered pig-to-human cardiac xenotransplantation: a case report. Lancet.

[73] Griffith BP (2022). Genetically modified porcine-to-human cardiac xenotransplantation. N Engl J Med.

[74] Moazami N (2023). Pig-to-human heart xenotransplantation in two recently deceased human recipients. Nat Med.

[75] Wang L, Cooper DKC, Burdorf L, Wang Y, Iwase H (2018). Overcoming coagulation dysregulation in pig solid organ transplantation in nonhuman primates: recent progress. Transplantation.

[76] Baldan N, Rigotti P, Calabrese F, Cadrobbi R, Dedja A, Iacopetti I, Boldrin M, Seveso M, Dall'Olmo L, Frison L, De Benedictis G, Bernardini D, Thiene G, Cozzi E, Ancona E (2004). Ureteral stenosis in HDAF Pig-to-primate renal xenotransplantation: a phenomenon related to immunological events?. Am J Transplant.

[77] Higginbotham L, Mathews D, Breeden CA, Song M, Farris AB, Larsen CP, Ford ML, Lutz AJ, Tector M, Newell KA, Joseph Tector A, Adams AB (2015). Pre-transplant antibody screening and anti-CD154 costimulation blockade promote long-term xenograft survival in a pig-to-primate kidney transplant model. Xenotransplantation.

[78] Iwase H, Liu H, Wijkstrom M, Zhou H, Singh J, Hara H, Ezzelarab M, Long C, Klein E, Wagner R, Phelps C, Ayares D, Shapiro R, Humar A, Cooper DKC (2015). Pig kidney graft survival in a baboon for 136days: longest life-supporting organ graft survival to date. Xenotransplantation.

[79] Kim SC, Mathews DV, Breeden CP, Higginbotham LB, Ladowski J, Martens G, Stephenson A, Farris AB, Strobert EA, Jenkins J, Walters EM, Larsen CP, Tector M, Tector AJ, Adams AB (2019). Long-term survival of pig-to-rhesus macaque renal xenografts is dependent on CD4 T cell depletion. Am J Transplant.

[80] Montgomery RA (2022). Results of two cases of pig-to-human kidney xenotransplantation. N Engl J Med.

[81] Porrett PM (2022). First clinical-grade porcine kidney xenotransplant using a human decedent model. Am J Transplant.

[82] Yu XH, D W, Jiang HT, Li T, Wang Y (2021). Kidney xenotransplantation: recent progress in preclinical research. Clin Chim Acta.

[83] Ekser B, Echeverri GJ, Hassett AC, Yazer MH, Long C, Meyer M, Ezzelarab M, Lin CC, Hara H, van der Windt DJ, Dons EM, Phelps C, Ayares D, Cooper DKC, Gridelli B (2010). Hepatic function after genetically engineered pig liver transplantation in baboons. Transplantation.

[84] Ekser B, Long C, Echeverri GJ, Hara H, Ezzelarab M, Lin CC, de Vera ME, Wagner R, Klein E, Wolf RF, Ayares D, Cooper DKC, Gridelli B (2010). Impact of thrombocytopenia on survival of baboons with genetically modified pig liver transplants: clinical relevance. Am J Transplant.

[85] Kim K, Schuetz C, Elias N, Veillette GR, Wamala I, Manish Varma R, Smith N, Robson SC, Benedict Cosimi A, Sachs DH, Hertl M (2012). Up to 9-day survival and control of thrombocytopenia following alpha1,3-galactosyl transferase knockout swine liver xenotransplantation in baboons. Xenotransplantation.

[86] Shah JA, Navarro-Alvarez N, DeFazio M, Rosales IA, Elias N, Yeh H, Colvin RB, Benedict Cosimi A, Markmann JF, Hertl M, Sachs DH, Vagefi PA (2016). A bridge to somewhere: 25-day survival after pig-to-baboon liver xenotransplantation. Ann Surg.

[87] Navarro-Alvarez N, Shah JA, Zhu A, Ligocka J, Yeh H, Elias N, Rosales I, Colvin R, Cosimi AB, Markmann JF, Hertl M, Sachs DH, Vagefi PA (2016). The effects of exogenous administration of human coagulation factors following pig-to-baboon liver xenotransplantation. Am J Transplant.

[88] Shah JA, Patel MS, Elias N, Navarro-Alvarez N, Rosales I, Wilkinson RA, Louras NJ, Hertl M, Fishman JA, Colvin RB, Cosimi AB, Markmann JF, Sachs DH, Vagefi PA (2017). Prolonged survival following pig-to-primate liver xenotransplantation utilizing exogenous coagulation factors and costimulation blockade. Am J Transplant.

[89] Harris DG, K Q, Dahi S, Burdorf L, Azimzadeh AM, Pierson RN (2015). Lung xenotransplantation: recent progress and current status. Xenotransplantation.

[90] Nguyen BN, Azimzadeh A, Zhang T, Wu G, Schuurman HJ, Sachs DH, Ayares D, Allan JS, Pierson RN (2007). Life-supporting function of genetically modified swine lungs in baboons. J Thor Cardiovasc Surg.

[91] Laird CT, Burdorf L, French BM, Kubicki N, Cheng X, Braileanu G, Sun W, O'Neill NA, Cimeno A, Parsell D, So E, Bähr A, Klymiuk N, Phelps CJ, Ayares D, Azimzadeh AM, Pierson RN (2017). Transgenic expression of human leukocyte antigen-E attenuates GalKO.hCD46 porcine lung xenograft injury. Xenotransplantation.

[92] Watanabe H, Sahara H, Nomura S, Tanabe T, Ekanayake-Alper DK, Boyd LK, Louras NJ, Asfour A, Danton MA, Ho S-H, Arn SJ, Hawley RJ, Shimizu A, Nagayasu T, Ayares D, Lorber MI, Sykes M, Sachs DH, Yamada K (2018). GalT-KO pig lungs are highly susceptible to acute vascular rejection in baboons, which may be mitigated by transgenic expression of hCD47 on porcine blood vessels. Xenotransplantation.

[93] Burdorf L, Azimzadeh AM, Pierson RN (2018). Progress and challenges in lung xenotransplantation: an update. Curr Opin Organ Transplant.

[94] Cooper DKC, Gaston R, Eckhoff D, Ladowski J, Yamamoto T, Wang L, Iwase H, Hara H, Tector M, Tector AJ (2018). Xenotransplantation-the current status and prospects. Br Med Bull.

[95] Hawthorne WJ, Lew AM, Thomas HE (2016). Genetic strategies to bring islet xenotransplantation to the clinic. Curr Opin Organ Transplant.

[96] Naqvi RA (2022). The future treatment for type 1 diabetes: pig islet- or stem cell-derived beta cells?. Front Endocrinol.

[97] Zhou Q (2022). Current status of xenotransplantation research and the strategies for preventing xenograft rejection. Front Immunol.

[98] Kemter E, Denner J, Wolf E (2018). Will genetic engineering carry xenotransplantation of pig islets to the clinic?. Curr Diab Rep.

[99] van der Windt DJ, Bottino R, Casu A, Campanile N, Smetanka C, He J, Murase N, Hara H, Ball S, Loveland BE, Ayares D, Lakkis FG, Cooper DKC, Trucco M (2010). Long-term controlled normoglycemia in diabetic non-human primates after transplantation with hCD46 transgenic porcine islets. Am J Transplant.

[100] Park C-G, Bottino R, Hawthorne WJ (2015). Current status of islet xenotransplantation. Int J Surg.

[101] Hawthorne WJ, Salvaris EJ, Phillips P, Hawkes J, Liuwantara D, Burns H, Barlow H, Stewart AB, Peirce SB, Hu M, Lew AM, Robson SC, Nottle MB, D’Apice AJF, O’Connell PJ, Cowan PJ (2014). Control of IBMIR in neonatal porcine islet xenotransplantation in baboons. Am J Transplant.

[102] Hara H, Cooper DKC (2011). Xenotransplantation-the future of corneal transplantation?. Cornea.

[103] Yoon CH, Choi HJ, Kim MK (2020). Corneal xenotransplantation: where are we standing?. Prog Retin Eye Res.

[104] Dong X, Hara H, Wang Y, Wang L, Zhang Y, Cooper DKC, Dai Y, Pan Z (2017). Initial study of α1,3-galactosyltransferase gene-knockout/CD46 pig full-thickness corneal xenografts in rhesus monkeys. Xenotransplantation.

[105] Cooper DKC, Satyananda V, Ekser B, van der Windt DJ, Hara H, Ezzelarab MB, Schuurman H-J (2015). Progress in pig-to-non-human primate transplantation models (1998–2013): a comprehensive review of the literature. Xenotransplantation.

[106] Zhang M-C, Liu X, Jin Y, Jiang D-L, Wei X-S, Xie H-T (2015). Lamellar keratoplasty treatment of fungal corneal ulcers with acellular porcine corneal stroma. Am J Transplant.

[107] Karnaukhova E (2013). C1-esterase inhibitor: biological activities and therapeutic applications. J Hematol Thromboembolic Dis.

[108] Caballero T (2021). Treatment of hereditary angioedema. J Investig Allergol Clin Immunol.

[109] Tillou X (2010). Recombinant human C1-inhibitor prevents acute antibody-mediated rejection in alloimmunized baboons. Kidney Int.

[110] Montgomery RA (2016). Plasma-derived C1 esterase inhibitor for acute antibody-mediated rejection following kidney transplantation: results of a randomized double-blind placebo-controlled pilot study. Am J Transplant.

[111] Vo AA (2015). A phase I/II placebo-controlled trial of C1-inhibitor for prevention of antibody-mediated rejection in HLA sensitized patients. Transplantation.

[112] Vogel C-W, Fritzinger D (2007). Humanized cobra venom factor: experimental therapeutics for targeted complement activation and complement depletion. Curr Pharm Design.

[113] Kock MA, Hew BE, Bammert H, Fritzinger DC, Vogel C-W (2004). Structure and function of recombinant cobra venom factor. J Biol Chem.

[114] Abicht J-M, Kourtzelis I, Reichart B, Koutsogiannaki S, Primikyri A, Lambris JD, Chavakis T, Holdt L, Kind A, Guethoff S, Mayr T (2016). Complement C3 inhibitor Cp40 attenuates xenoreactions in pighearts perfused with human blood. Xenotransplantation.

[115] Silasi-Mansat R, Zhu H, Popescu NI, Peer G, Sfyroera G, Magotti P, Ivanciu L, Lupu C, Mollnes TE, Taylor FB, Kinasewitz G, Lambris JD, Lupu F (2010). Complement inhibition decreases the procoagulant response and confers organ protection in a baboon model of *Escherichia coli* sepsis. Blood.

[116] Kourtzelis A, Ferreira I, Mitroulis D, Ricklin S, Bornstein C, Waskow J, Lambris TC (2015). Complement inhibition in a xenogeneic model of interactions between human whole blood and porcine endothelium. Horm Metab Res.

[117] Zhou H, Hara H, Cooper DKC (2019). The complex functioning of the complement system in xenotransplantation. Xenotransplantation.

[118] Shapiro R, Chin-Yee I, Lam S (2015). Eculizumab as a bridge to immunosuppressive therapy in severe cold agglutinin disease of anti-Pr specificity. Clin Case Rep.

[119] Hillmen P, Young NS, Schubert J, Brodsky RA, Socié G, Muus P, Röth A, Szer J, Elebute MO, Nakamura R, Browne P, Risitano AM, Hill A, Schrezenmeier H, Chieh-Lin F, Maciejewski J, Rollins SA, Mojcik CF, Rother RP, Luzzatto L (2006). The complement inhibitor eculizumab in paroxysmal nocturnal hemoglobinuria. N Engl J Med.

[120] Notaro R, Sica M (2018). C3-mediated extravascular hemolysis in PNH on eculizumab: mechanism and clinical implications. Semin Hematol.

[121] Ricklin D, Mastellos DC, Reis ES, Lambris JD (2018). The renaissance of complement therapeutics. Nat Rev Nephrol.

[122] Ricklin D, Lambris JD (2016). New milestones ahead in complement-targeted therapy. Semin Immunol.

[123] Risitano AM, Marotta S (2016). Therapeutic complement inhibition in complement-mediated hemolytic anemias: past, present and future. Semin Immunol.

[124] Paul Morgan B, Harris CL (2015). Complement, a target for therapy in inflammatory and degenerative diseases. Nat Rev Drug Discov.

[125] Agostinis C, Balduit A, Mangogna A, Zito G, Romano F, Ricci G, Kishore U, Bulla R (2021). Immunological basis of the endometriosis: the complement system as a potential therapeutic target. Front Immunol.

[126] Zhou Y, Zhou S, Wang Q, Zhang B (2024). Mitigating cross-species viral infections in xenotransplantation: progress, strategies, and clinical outlook. Cell Transplant.

